# The relationship between children’s oral health-related behaviors and their caregiver’s social support

**DOI:** 10.1186/s12903-016-0270-4

**Published:** 2016-09-01

**Authors:** Rong Min Qiu, Ye Tao, Yan Zhou, Qing Hui Zhi, Huan Cai Lin

**Affiliations:** 1 Department of Preventive Dentistry, Guanghua School of Stomatology, Guangdong Provincial Key Laboratory of Stomatology, Sun Yat-sen University, 56 Ling Yuan Road West, 510055 Guangzhou, Guangdong Province China; 2 Department of Pediatric Dentistry, College of Stomatology, Guangxi Medical University, Nanning, China

**Keywords:** Child, Caregiver, Social support, Oral health-related behavior, Psychosocial factor

## Abstract

**Background:**

Social support might play a role in helping people adopt healthy behaviors and improve their health. Stronger social support from mothers has been found to be positively related to higher tooth brushing frequency in 1- to 3-year-old children. However, little is known regarding the relationship between the caregiver’s social support and the oral health-related behaviors of 5-year-old children in China. This study aimed to investigate this relationship.

**Methods:**

A cross-sectional study was conducted among 1332 5-year-old children and their caregivers in Guangzhou, southern China. Data were collected using questionnaires that were completed by the caregivers and the children’s caries status were examined. The caregivers’ social support was measured using the Social Support Rating Scale. The measurements of the children’s oral health-related behaviors included the frequencies of sugary snack intake and tooth brushing, utilization of dental services, and patterns of dental visits. Univariate and multiple logistic regression analyses were used to analyze the relationships between the variables.

**Results:**

No association was found between the caregiver’s social support and the child’s oral health-related behaviors in a multiple logistic regression analysis. However, other factors, particularly the oral health-related behaviors of the caregiver, were found to be significantly linked to the child’s oral health-related behaviors.

**Conclusions:**

The oral health-related behaviors of 5-year-old children in Guangzhou are unrelated to the caregiver’s social support but are related to other specific factors, particularly the caregiver’s oral health-related behaviors.

## Background

Despite great improvements in the oral health of people in many countries over the past few decades, dental caries in preschool children are a public health problem of great concern [1]. The occurrence of dental caries is strongly related to oral health behaviors, such as sugar consumption, tooth brushing habits and dental care [1, 2]. Understanding the factors that affect oral health behaviors is crucial for the development of programs to prevent dental caries.

Researchers studying dental health have documented the need to understand the role of oral health knowledge and attitudes in the oral health behaviors of individuals [3, 4]. However, a shift of focus toward the psychosocial determinants of oral health behaviors has become evident [5, 6]. Social support is a psychosocial factor that might help individuals cope with psychological distress, adopt healthy behaviors and improve their health [7, 8].

Social support is defined as “assistance and protection given to others, especially to individuals” [9]. It can essentially be divided into two categories [10]. The first is objective, visible or actual support, which reflects the material support that individuals might directly receive, and the social network or the community that individuals belong to. The second is subjective and emotional support, which reflects the emotional experience and degree of satisfaction experienced when individuals feel respected, supported and understood. When individuals receive more social support, they may be better able to cope with psychological distress in their life, which could help them maintain better health. It has been reported that those who receive less social support or use their available social support less effectively are more likely to adopt unhealthy oral health behaviors, such as smoking and alcohol consumption, which could increase their risk of developing periodontitis [11].

Social support of children is provided by their caregivers. Access to and availability of social support from their caregivers could affect the oral health of children. Children from families with better access to social support are more likely to utilize dental services, have better oral health habits, and exhibit better oral health [12–14]. A mother’s social support is significantly associated with the tooth brushing habits of children aged 1-3 years but not with those of children aged 4-5 years [15]. The reason for this might be that children younger than 3 years have no ability to live independently and are totally dependent on their caregivers. Thus far, little is known regarding the relationship between the social support of caregivers and the oral health-related behaviors of their children in China.

Our study is a further step in the exploration of the relationship between the social support of caregivers and the oral health-related behaviors of 5-year-old children in China. We hypothesized that a caregiver with more social support would have a child with better oral health-related behaviors, such as a lower frequency of sugary snack intake, more frequent tooth brushing and utilization of dental care services, and regular dental check-ups, than a caregiver with less social support.

## Methods

This cross-sectional study was carried out from August 2011 to December 2011 in Guangzhou, China.

### Study population

The target population of this study was 5-year-old children and their main caregivers in Guangzhou, China. The main caregiver was defined as the individual who was primarily in charge of the child on a day-to-day basis. For the purposes of this study, the main caregiver could be the child’s mother, father or grandparent.

### Sampling method

The sampling method was described in our previous study [16]. The sample size was calculated based on the prevalence of dental caries among 5-year-old children in Guangzhou, as reported in the third Chinese national oral health survey [17]. A standard error of 1.5 % was set as the precision of the estimate. Finally, a minimum sample size of 1067 children was calculated as the number needed to satisfy the requirements. The selection of participants was based on a two-stage area probability sample of the 12 administrative districts in Guangzhou. In the first stage, six randomly selected districts were the primary sampling units. In the second stage, four kindergartens were randomly selected in each of the selected administrative districts, and all of the 5-year-old children in the selected kindergartens and their main caregivers were invited to take part in the study. All the caregivers were fully informed of the study purpose in writing, and participation in the study was voluntary.

### Instruments and measures

Information relating to the following data was collected using a questionnaire administered to the caregivers: the child’s oral health-related behaviors, the caregiver’s social support, the caregiver’s oral health knowledge, the caregiver’s oral health attitudes, the caregiver’s oral health-related behaviors, the demographic and socioeconomic background of the participants. The questionnaire was distributed by the kindergarten teachers to the caregivers, who completed the questionnaire at home and returned it to the kindergarten teachers.

#### Oral health-related behaviors of children and caregivers

The oral health-related behaviors of 5-year-old children and their caregivers that were assessed in this study included the frequency of sugary snack intake (<once/day vs. ≥once/day), tooth brushing frequency (≤once/day vs. ≥twice/day), having used dental services ever (yes vs. no), and the pattern of dental visits for those children who had visited a dentist (for the treatment of dental problems vs. mainly for check-ups). The design of the oral health-related behavior assessment was described in our previous study [16].

#### Social support

Social support was measured via the Social Support Rating Scale (SSRS) proposed by Xiao [18]. The SSRS contains 10 items and measures the following dimensions of social support: objective support (3 items), subjective support (4 items), and the availability of support (3 items). Objective support reflects the degree of actual support that an individual received in the past, subjective support reflects the perceived interpersonal network that an individual can count on, and the availability of support refers to the pattern of behavior that an individual utilizes when seeking social support. The scores of the 10 items were summed to obtain the overall SSRS score. Thus, the SSRS score could range from 12 to 66, with higher scores indicating stronger social support. Objective support scores ranged from 1 to 22, subjective support scores ranged from 8 to 32, and the support availability scores ranged from 3 to 12. Cultural adaptation of the SSRS has been undertaken in China. The SSRS has been applied in a wide range of Chinese people and has been shown to have good reliability and validity [19, 20]. In this sample, the *Cronbach*’s alpha coefficient of the SSRS was 0.76.

#### Oral health knowledge and oral health attitudes of the caregivers

The oral health knowledge of the caregivers was measured using four questions regarding the causes and prevention of dental caries and periodontal disease, which were used in a previous study of adults in Guangdong [21]. The two questions relating to the causes and prevention of dental caries were as follows: “Which of the following statements do you believe describe a cause of dental caries?” and “Which of the following statements do you believe describe a method to prevent dental caries?”. The two questions relating to the causes and prevention of periodontal disease were as follows: “Which of the following statements do you believe describe a cause of periodontal disease?” and “Which of the following statements do you believe describe a method to prevent periodontal disease?”. A score of 1 was given for each correct answer to a question, and a score of 0 was given for an incorrect answer or for an answer of “I don’t know”. For each question, there were only four correct answers, and the maximum score was 4. The scores from the four questions were summed to obtain the overall oral health knowledge score, which could range from 0 to 16, with higher scores indicating better oral health knowledge.

To explore the attitudes of caregivers toward oral health, eight questions on dental health beliefs and the importance of oral health, retaining natural teeth, and the use of dental service were used, which were used in a previous study [22]. There were three possible responses to each statement: “agree,” “disagree” or “neither”. For each question, caregivers showing a positive attitude received a score of 1. The total scores for dental attitude were calculated by summing the scores of the eight questions. The final scores ranged from 0 to 8, with higher scores indicating a more positive attitude toward oral health.

#### Demographic and socioeconomic background

The demographic data included the gender of the child, single-child status, marital status of the parents, caregiver type and socioeconomic background data, including the mother’s education and occupation, father’s education and occupation, family income, and the child’s dental insurance.

### Clinical examination

Assessments of the caries status of each child who returned a fully answered questionnaire were carried out by a trained examiner. Decayed, missing and filled teeth (dmft) were recorded according to the recommended criteria of the World Health Organization [23]. The dmft value was set as one of the factors related to the child’s dental service use and his/her pattern of dental visits.

### Statistical analysis

Data analysis was conducted with SPSS for Windows (version 16.0, Chicago, IL, USA). In the analysis, the child’s oral health-related behaviors were set as the outcomes, and the caregiver’s social support was set as the independent variable. The caregiver’s oral health knowledge and attitudes, the caregiver’s oral health-related behaviors, and the child’s demographic background, socioeconomic background and dmft value were set as the controlling variables.

The total SSRS score was analyzed as a continuous variable. First, in the univariate analysis, two-sample *t*-tests were used to analyze the relationship between the SSRS scores and the outcome variables, and the relationships between the controlling variables and the outcome variables were assessed via chi-square tests or *t*-tests. The controlling variables in the univariate analysis with a *p* value not higher than 0.20 were included in the multiple logistic regression analysis. Second, the effects of the independent variable and the controlling variables on the outcome variables were tested via multiple logistic regression analysis. For all of the statistical tests, a *p* value of 0.05 was set as the level of significance.

## Results

In total, 1440 questionnaires were distributed; of these, 1332 were fully completed and returned, 46 were not returned, and 62 were returned incomplete and with most items unanswered. Thus, the data from 1332 caregivers (1141 mothers, 85.7 %; 72 fathers, 5.4 %; and 119 grandparents, 8.9 %) were used for the analysis of the relationship between the caregiver’s social support and the child’s oral health-related behaviors. Among the children of the 1332 caregivers, 54.9 % (731) were boys and 45.1 % (601) were girls.

The frequency distributions of the categorical variables and the values of the continuous variables are shown in Tables 1 and 2.

**Table 1:** Frequency distribution of the categorical variables

Variables	Number	Percentage
Gender		
Boy	731	54.9
Girl	601	45.1
Single child		
Yes	936	70.3
No	396	29.7
Caregivers		
Mother	1141	85.7
Father	72	5.4
Grandparent	119	8.9
Marital status of parents		
Cohabiting	1309	98.3
Not cohabiting	23	1.7
Mother’s education		
≤ high school graduated	727	54.6
≥ university graduated	605	45.4
Mother’s occupation		
Unemployed	248	18.6
Employee/non-professional	794	59.6
Employer/professional	290	21.8
Father’s education		
≤ high school graduated	677	50.8
≥ university graduated	655	49.2
Father’s occupation		
Unemployed	52	3.9
Employee/non-professional	846	63.5
Employer/professional	434	32.6
Family monthly income (per-capital)		
< 2000 RMB	262	19.7
2000-4999 RMB	546	41
≥ 5000 RMB	524	39.3
Child’s dental insurance		
No	1056	79.3
Yes	276	20.7
Child’s frequency of sugary snack intake		
< once/day	671	50.4
≥ once/day	661	49.6
Child’s toothbrushing frequency		
≤ once/day	896	67.3
≥ twice/day	436	32.7
Child’s dental service utilization		
No	923	69.3
Yes	409	30.7
Child’s pattern of dental attendance		
For treatment of dental problem	348	85.1
Mainly for check-up	61	14.9
Caregiver’s frequency of sugary snack intake		
< once/day	833	62.5
≥ once/day	499	37.5
Caregiver’s toothbrushing frequency		
≤ once/day	405	30.4
≥ twice/day	927	69.6
Caregiver’s dental service utilization		
No	381	28.6
Yes	951	71.4
Caregiver’s pattern of dental attendance		
For treatment of dental problem	833	87.6
Mainly for check-up	118	12.4

**Table. 2:** Values of the continuous variables

Variables	Mean	SD
Caregiver’s social support	43.2	6.6
Caregiver’s oral health knowledge	9.1	3.9
Caregiver’s oral health attitudes	6.2	1.4
Child’s dmft value	3.8	4.5

The caregivers’ total SSRS scores ranged from 19 to 62, with a mean of 43.2 and a standard deviation of 6.6. The means and standard deviations of the SSRS scores of the different types of caregivers were, respectively, 43.4 and 6.6 for the mothers, 41.8 and 6.7 for the fathers, and 42.6 and 6.6 for the grandparents. No statistically significant differences were found in the total SSRS scores among the various types of caregivers (*p* = 0.083).

The results show that the caregiver’s social support was significantly associated with the child’s tooth brushing frequency, utilization of dental care, and pattern of dental visits but not with the frequency of sugary snack intake. Children whose caregivers had higher SSRS scores had a higher frequency of tooth brushing (*p* = 0.007) and utilization of dental care (*p* = 0.006) than did the children whose caregivers scored lower on the SSRS. Among the children having received dental care, the children whose caregivers scored higher on the SSRS were more likely to visit the dentist mainly for check-ups than for treatment of a dental problem (*p* = 0.009) (Table 3).

**Table 3:** Univariate analysis between the child's oral health-related behaviors and the caregiver's total social support (*n* = 1332)

Outcome variables	Number	Percentage	Social support	*P-*value
			Mean (SD)	
Child’s frequency of sugary snack intake				
< once/day	671	50.4	43.4 (6.5)	0.434
≥ once/day	661	49.6	43.1 (6.7)	
Child’s toothbrushing frequency				
≤once/day	896	67.3	42.9 (6.7)	**0.007**
≥twice/day	436	32.7	43.9 (6.4)	
Child’s utilization of dental care of children				
No	923	69.3	42.9 (6.7)	**0.006**
Yes	409	30.7	44.0 (6.2)	
Child’s pattern of dental attendance				
For treatment of dental problem	348	85.1	43.7 (6.3)	**0.009**
Mainly for check-up	61	14.9	45.9 (5.9)	

A *p* value of 0.05 was set as the level of significance. Bold data is a *p* value set as the level of significance

Because the caregiver’s social support was associated with the child’s tooth brushing frequency, utilization of dental care, and pattern of dental visits, a further analysis was performed.

### Social support and children’s tooth brushing frequency

As shown in Table 4, it was found that the controlling variables of a child’s gender, single-child status, type of caregiver, mother’s education and occupation, father’s education, family monthly income, caregiver’s tooth brushing frequency, and caregiver’s oral health knowledge and attitudes were significantly associated with the child’s tooth brushing frequency.

**Table 4:** Univariate analysis between the control variables and the child’s toothbrushing frequency (*n* = 1332)

Variables	Frequency of toothbrushing		
	≤once/day	≥twice/day	*P-*value
	n (%)	n (%)	
Gender			
Boy	513 (57.3)	218 (50.0)	
Girl	383 (42.7)	218 (50.0)	0.013*
Single child			
Yes	600 (67.0)	336 (77.1)	
No	296 (33.0)	100 (22.9)	<0.001*
Caregivers			
Mother	755 (84.3)	386 (88.5)	
Father	53 (5.9)	19 (4.4)	
Grandparent	88 (9.8)	31 (7.1)	0.113*
Marital status of parents			
Cohabiting	878 (98.0)	431 (98.9)	
Not cohabiting	5 (2.0)	18 (1.1)	0.257*
Mother’s education			
≤ high school graduated	545 (60.8)	182 (41.7)	
≥ university graduated	351 (39.2)	254 (58.3)	<0.001*
Mother’s occupation			
Unemployed	180 (20.1)	68 (15.6)	
Employee/non-professional	543 (60.6)	251 (57.6)	
Employer/professional	173 (19.3)	117 (36.8)	0.004*
Father’s education			
≤ high school graduated	509 (56.8)	168 (38.5)	
≥ university graduated	387 (43.2)	268 (61.5)	<0.001*
Father’s occupation			
Unemployed	37 (4.1)	15 (3.4)	
Employee/non-professional	585 (65.3)	261 (59.9)	
Employer/professional	274 (30.6)	160 (36.7)	0.079*
Family monthly income (per-capital)			
< 2000 RMB	195 (21.8)	67 (15.4)	0.002*
2000-4999 RMB	374 (41.7)	172 (39.4)	
≥ 5000 RMB	327 (36.5)	197 (45.2)	
Caregiver’s toothbrushing frequency			
≤ once/day	346 (38.6)	59 (13.5)	
≥ twice/day	550 (61.4)	377 (86.5)	<0.001*
	Mean(SD)	Mean(SD)	
Caregiver’s oral health knowledge score	8.7 (4.0)	9.9 (3.5)	<0.001**
Caregiver’s oral health attitude score	6.2 (1.4)	6.4 (1.3)	0.010**

(*by Chi-square test; **by *t*-test). A *p* value of 0.05 was set as the level of significance

Bold data is a *p* value set as the level of significance

After adjusting for the above controlling variables in a multiple logistic regression analysis, the caregiver’s SSRS score was not significantly associated with the child’s tooth brushing frequency. The child’s tooth brushing frequency was significantly related to the child’s sex, mother’s and father’s education, and caregiver’s tooth brushing frequency and oral health knowledge. Girls were more likely to brush their teeth twice or more a day (OR = 1.30, *p* = 0.034). Children with a mother or father with a higher level of education brushed their teeth more frequently (OR = 1.39, *p* = 0.035; OR = 1.39, *p* = 0.031, respectively). Children whose caregivers brushed their teeth more frequently were more likely to brush their teeth more frequently (OR = 3.57, *p* < 0.001) (Table 5).

**Table 5:** Multiple logistic regression analysis between the caregiver's social support and the child’s toothbrushing frequency (*n* = 1332)

Variables	Frequency of toothbrushing	
	(≤once/day vs. ≥ twice/day)*	
	Adjusted OR	*P-*value
	(95 % CI)	
Caregiver’s social support	-	**-**
Gender		
Boy	1	
Girl	1.30 (1.02 ~ 1.67)	**0.034**
Single child		
Yes	1	
No	0.77 (0.58 ~ 1.02)	0.072
Caregivers		
Mother		
Father	-	-
Grandparent	-	-
Mother’s education		
≤ high school graduated	1	
≥ university graduated	1.39 (1.02 ~ 1.88)	**0.035**
Mother’s occupation		
Unemployed		
Employee/non-professional	-	-
Employer/professional	-	-
Father’s education		
≤ high school graduated	1	
≥ university graduated	1.39 (1.03 ~ 1.87)	**0.031**
Father’s occupation		
Unemployed		
Employee/non-professional	-	-
Employer/professional	-	-
Family monthly income (per-capital)		
< 2000 RMB		
2000-4999 RMB	-	-
≥ 5000 RMB	-	-
Caregiver’s toothbrushing frequency		
≤ once/day	1	
≥ twice/day	3.57 (2.61 ~ 4.87)	**<0.001**
Caregiver’s oral health knowledge score	1.04 (1.01 ~ 1.07)	**0.03**
Caregiver’s oral health attitude score	-	

*in the multiple logistic regression analysis, “≤once/day” was set as the reference category. A *p *value of 0.05 was set as the level of significance

Bold data is a *p* value set as the level of significance

### Social support and children’s use of dental care

As shown in Table 6, the controlling variables of the father’s occupation, caregiver’s utilization of dental care and the child’s dmft value were significantly associated with the child’s utilization of dental care.

**Table 6:** Univariate analysis between the control variables and the child’s utilization of dental care (*n* = 1332)

Variables	Utilization of dental care		
	No	Yes	*P-*value
	n (%)	n (%)	
Gender			
Boy	514 (55.7)	217 (53.1)	
Girl	409 (44.3)	192 (46.9)	0.373*
Single child			
Yes	638 (69.1)	298 (72.9)	
No	285 (30.9)	111 (27.1)	0.169*
Caregivers			
Mother	783 (84.8)	358 (87.5)	
Father	51 (5.5)	21 (5.2)	
Grandparent	89 (9.7)	30 (7.3)	0.366*
Marital status of parents			
Cohabiting	905 (98.0)	404 (98.8)	
Not cohabiting	18 (2.0)	5 (1.2)	0.347*
Mother’s education			
≤ high school graduated	505 (54.7)	222 (54.3)	
≥ university graduated	418 (45.3)	187 (45.7)	0.883*
Mother’s occupation			
Unemployed	180 (19.5)	68 (16.6)	
Employee/non-professional	533 (57.8)	261 (63.8)	
Employer/professional	210 (22.8)	80 (19.6)	0.114*
Father’s education			
≤ high school graduated	470 (50.9)	207 (50.6)	
≥ university graduated	453 (49.1)	202 (49.4)	0.917*
Father’s occupation			
Unemployed	45 (4.8)	7 (1.7)	
Employee/non-professional	584 (63.3)	262 (64.1)	
Employer/professional	294 (31.9)	140 (34.2)	0.02*
Family monthly income (per-capital)			
< 2000 RMB	184 (19.9)	78 (19.1)	0.929*
2000-4999 RMB	378 (41.0)	168 (41.1)	
≥ 5000 RMB	361 (39.1)	163 (39.9)	
Child’s dental insurance			
No	719 (77.9)	337 (82.4)	
Yes	204 (22.1)	72 (17.6)	0.062*
Caregiver’s dental insurance			
No	676 (73.2)	303 (74.1)	
Yes	247 (26.8)	106 (25.9)	0.748*
Caregiver’s utilization of dental care			
No	295 (32.0)	86 (21.0)	
Yes	628 (68.0)	323 (79.0)	<0.001*
	Mean(SD)	Mean(SD)	
Caregiver’s oral health knowledge score	9.0 (3.8)	9.3 (4.0)	0.258**
Caregiver’s oral health attitude score	6.2 (1.4)	6.3 (1.4)	0.628**
dmft	3.0 (4.0)	5.4 (5.2)	<0.001**

(*by Chi-square test; **by t-test). A *p* value of 0.05 was set as the level of significance

Bold data is a *p* value set as the level of significance

After adjusting for the above controlling variables in a multiple logistic regression analysis, the caregiver’s SSRS score was not significantly associated with the child’s utilization of dental care. The child’s utilization of dental care was significantly associated with the father’s occupation, caregiver’s utilization of dental care and child’s dmft value. Children with more caries, children whose father was an employer/professional, and children whose caregiver had used dental care were more likely to have visited a dentist (all *p* < 0.05) (Table 7).

**Table 7:** Multiple logistic regression analysis between the caregiver's social support and children’s the child's utilization of dental care (*n* = 1332)

Variables	Utilization of dental care	
	(No vs. Yes)*	
	Adjusted OR	*P-*value
	(95 % CI)	
Caregive's social support	1.02(0.99 ~ 1.04)	0.094
Single child		
Yes		
No	-	**-**
Mother’s occupation		
Unemployed	1	0.082
Employee/non-professional	1.01 (0.79 ~ 1.54)	0.575
Employer/professional	0.74 (0.48 ~ 1.14)	0.171
Father’s occupation		**0.025**
Unemployed		
Employee/non-professional	2.67 (1.14 ~ 6.23)	**0.023**
Employer/professional	3.35 (1.40 ~ 7.98)	**0.006**
Child’s dental insurance		
No		
Yes	-	**-**
Caregiver’s utilization of dental care		
No	1	
Yes	1.75 (1.31 ~ 2.33)	**<0.001**
dmft	1.12 (1.09 ~ 1.15)	**<0.001**

*in the multiple logistic regression analysis, “No” was set as the reference category. A *p* value of 0.05 was set as the level of significance

Bold data is a *p* value set as the level of significance

### Social support and children’s patterns of dental visits

As shown in Table 8, the controlling variables of family monthly income, a caregiver’s pattern of dental visits and oral health attitude, and a child’s dmft value were significantly associated with the pattern of a child’s dental visits.

**Table 8:** Univariate analysis between the control variables and the child’s pattern of dental attendance (*n* = 409)

Variables	Pattern of dental attendance		
	For treatment of dental problem	Mainly for check-up	*P-*value
	n (%)	n (%)	
Gender			
Boy	185 (55.2)	32 (52.5)	0.919*
Girl	163 (46.8)	29 (47.5)	
Single child			
Yes	251 (72.1)	47 (77.0)	0.425*
No	97 (27.9)	14 (23.0)	
Caregiver			
Mother	303 (87.1)	55 (90.2)	0.728*
Father	18 (5.2)	3 (4.9)	
Grandfather or grandmother	27 (7.7)	3 (4.9)	
Marital status of parents			
Cohabiting	193 (55.5)	659 (98.2)	0.862*
Not cohabiting	11 (1.7)	12 (1.8)	
Mother’s education			
≤ high school graduated	193 (55.5)	29 (47.5)	0.252*
≥ college graduated	155 (44.5)	32 (52.5)	
Mother’s occupation			
Unemployed	57 (16.4)	11 (18.0)	0.478*
Employee/non-professional	226 (64.9)	35 (57.4)	
Employer/professional	65 (18.7)	15 (24.6)	
Father’s education			
≤ high school graduated	183 (52.6)	24 (39.3)	0.056*
≥ college graduated	165 (47.4)	37 (60.7)	
Father’s occupation			
Unemployed	7 (2.0)	0 (0)	0.202*
Employee/non-professional	227 (65.2)	35 (57.4)	
Employer/professional	114 (32.8)	26 (42.6)	
Family monthly income (per-capital)			
< 2000 RMB	69 (19.8)	9 (14.8)	0.048*
2000-4999 RMB	149 (42.8)	19 (31.1)	
≥ 5000 RMB	130 (37.4)	33 (54.1)	
Child’s dental insurance			
No	293 (84.2)	44 (72.1)	0.022*
Yes	55 (15.8)	17 (27.9)	
Caregiver’s pattern of dental attendance			
For treatment of dental problem	245 (90.4)	31 (59.6)	<0.001*
Mainly for check-up	26 (9.6)	21 (40.4)	
	Mean(SD)	Mean(SD)	
Caregiver’s oral health knowledge score	9.2 (4.0)	100 (3.8)	0.117**
Caregiver’s oral health attitude score	6.2 (1.4)	6.6 (1.3)	0.027**
Child'sdmft value	5.7 (5.3)	3.6 (4.4)	0.005**

(* by Chi-square test; ** by t-test). A *p* value of 0.05 was set as the level of significance

Bold data is a *p* value set as the level of significance

After adjusting for the above controlling variables in a multiple logistic regression analysis, the caregiver’s SSRS score was not significantly linked to the child’s utilization of dental care. The pattern of a child’s dental care utilization was significantly associated with the pattern of the caregiver’s dental care utilization and the dmft value of the child. Caregivers who visited the dentist primarily for check-ups had children who visited the dentist primarily for check-ups (OR = 5.78, *p* < 0.001). A child who had more teeth caries was not likely to have visited the dentist primarily for check-ups but rather for the treatment of dental problems (OR = 0.93, *p* = 0.035) (Table 9).

**Table 9:** Multiple logistic regression analysis between the caregiver's social support and children’s and the child's pattern of dental attendance (*n* = 409)

Variables	Pattern of dental attendance	
	(For treatment of dental problem vs. Mainly for check-up)*	
	Adjusted OR	*P-*value
	(95 % CI)	
Caregive's social support	-	-
Father’s education		
≤ high school graduated		
≥ college graduated	-	-
Family monthly income (per-capital)		
< 2000 RMB		
2000-4999 RMB	-	-
≥ 5000 RMB	-	-
Children’s dental insurance		
No		
Yes	-	-
Caregiver’s pattern of dental attendance		
For treatment of dental problem		
Mainly for check-up	5.78 (2.89-11.6)	**<0.001**
Caregiver’s oral health attitude score	-	-
Child's dmft value	0.93 (0.87-0.99)	**0.035**

*in the multiple logistic regression analysis, “for treatment of dental problem” was set as the reference category. A *p* value of 0.05 was set as the level of significance

Bold data is a *p* value set as the level of significance

## Discussion

This cross-sectional study shows that the associations between a caregiver’s social support and a child’s oral health-related behaviors, including the frequency of sugary snack intake, tooth brushing frequency, use of dental services and pattern of dental visits, were not significant.

In this study, the tooth brushing frequency of 5-year-old children was not related to the social support of their caregivers. This result is similar to that of a previous study [15]. The brushing frequency of 4-5-year-old children was not associated with their mother’s social support and was positively associated with higher family income and dental insurance coverage; higher family income and improved insurance coverage could help increase access to needed services and foster dental health-promoting habits [15]. In our study, a child’s tooth brushing frequency was linked to other factors, including the child’s sex, the mother and father’s education, and the caregiver’s tooth brushing frequency and oral health knowledge. These results indicate that caregivers who were more knowledgeable regarding oral hygiene needs and who brushed their own teeth more frequently had children who brushed more frequently. Girls were found to brush their teeth more frequently, and it is possible that the girls received more attention from the caregivers than the boys. This result indicates that boys should receive more attention from programs aimed at promoting oral health behaviors in Guangzhou, China.

Research has shown that mothers with greater social support received more information regarding dental care from family members or friends and focused more on improving access to dental care for their younger children [14]. However, in our study, no significant association between the caregiver’s social support and the child’s utilization of dental care was found; the same result was found for the pattern of a child’s utilization of dental care. In our study, approximately 30.7 % of the children had received dental care; only 14.9 % of the children had visited the dentist primarily for check-ups. We found that the children with more caries were more likely to have had dental visits and to have visited the dentist predominantly for treatment of dental problems. These findings indicate that the occurrence of dental caries was the principal reason for children to visit a dentist. Additionally, our study revealed that a child’s utilization of dental care and their pattern of dental care use were strongly linked to the caregiver’s utilization of dental care and pattern of dental care use, respectively. Most of the caregivers had dental visits that were predominantly for dental problems, indicating that caregivers neglect the importance of regular dental check-ups in their own and their children’s oral health. The severe shortage of pediatric dentists was a limitation leading to the lack of utilization of dental care for children and the lack of information and assistance provided to caregivers by dentists.

No research has been conducted to explore the relationship between a caregiver’s social support and a child’s frequency of sugary snack intake. In this study, the caregiver’s social support was not significantly associated with a child’s frequency of sugary snack intake. The frequency of sugary snack intake by children was predominantly related to the caregiver’s frequency of sugary snack intake and other factors, which was also shown in our previous study [16, 22]. In addition, these findings revealed that a child’s sugary snack intake habits are influenced by his/her caregiver’s sugary snack intake habits.

In Guangzhou, China, a child’s oral health-related behaviors were associated with his/her caregiver’s oral health-related behaviors and not with his/her caregiver’s social support. The reason that the social support of the caregiver was not related to the child’s oral health-related behaviors is not clear. Other research has shown that the social support of individuals is associated with their oral health-related behaviors [7, 8]. In the present study, the caregiver’s oral health-related behaviors were related to the child’s oral health-related behaviors; the social support of a caregiver could indirectly influence a child’s oral health-related behaviors through influences on the caregiver’s own oral health-related behaviors. This is a hypothesis that needs further study.

Some further points should be noted with respect to this study. Adoption of consistent behavioral habits in childhood occurs at home, and caregivers could be the primary models for children’s behavior. Caregivers with good personal oral hygiene skills are more likely to understand the importance of a child’s oral health than caregivers with poor oral hygiene skills and would likely be more effective in controlling proper tooth brushing, sugary snack intake and other oral health behaviors of their children [24, 25]. We have demonstrated that caregivers who have better oral health habits are more likely to have children with better oral health habits. Young children depend on their caregivers to take care of their oral health needs, and caregivers play a key role in influencing the habits and health status of their children. Therefore, more attention should be paid to improving the oral health-related behaviors of caregivers for the promotion of children’s oral health.

This study was the first to explore the relationship between the social support of caregivers and their children’s oral health behaviors, and it has some limitations. First, the data in this study were cross-sectional, which precludes drawing inferences regarding the causal relationships between the social support of caregivers and the oral health behaviors of their children. Further work on this topic should adopt a longitudinal approach. Second, further research should be conducted to explore whether the social support of a caregiver might influence his/her oral health-related behaviors, thus indirectly influencing his/her child’s oral health-related behaviors.

## Conclusions

These findings indicate that the oral health-related behaviors of 5-year-old children in Guangzhou are unrelated to the social support of their caregivers and are instead related to other specific factors, particularly the caregiver’s oral health-related behaviors. The potential for caregivers to play a significant role in the oral health of their children should be considered in the development of programs to promote oral health.

## Abbreviations

dmft: decayed, missing and filled teeth
SSRS: Social support rating scale
